# ﻿*Neilliadaloushanensis*, a new species of *Neillia* (Rosaceae) from southwest China

**DOI:** 10.3897/phytokeys.261.152449

**Published:** 2025-08-15

**Authors:** Xu Wu, Dai-Kun Ma, Ming-Tai An, Yu-Cai Feng, Jiang-Hong Yu, Ji-Huai Han

**Affiliations:** 1 College of Forestry, Guizhou University, Guiyang 550025, Guizhou, China; 2 Key Laboratory of Systematic and Evolutionary Botany/State Key Laboratory of Plant Diversity and Specialty Crops, Institute of Botany, Chinese Academy of Sciences, Beijing 100093, China; 3 University of Chinese Academy of Sciences, Beijing 100049, China; 4 Guizhou Dashahe National Nature Reserve Administration, Daozhen 563500, Guizhou, China; 5 Key Laboratory of Plant Resource Conservation and Germplasm Innovation in Mountainous Region (Ministry of Education), College of Life Sciences/Institute of Agro-bioengineering, Guizhou University, Guiyang 550025, Guizhou, China

**Keywords:** Guizhou, *Neillia*, new species, phylogenomic, Rosaceae, phylogenomic

## Abstract

Through detailed morphological comparison and robust molecular evidence, we confirm *Neilliadaloushanensis* M.T.An & Xu Wu as a new species of Rosaceae from Guizhou province, southwest China. Morphologically, *N.daloushanensis* closely resembles *N.gracilis* but differs in several key traits: it is a creeping liana rather than a low subshrub; its stems are 0.6–1.5 cm long compared to the slender stems less than 0.5 cm tall in *N.gracilis*; its leaves are larger, measuring 5–9 × 3–6 cm vs. 2.5–3.5 × 2–3 cm; stipules are sessile, clasping, and larger (0.8–1.3 × 0.6–1.1 cm) rather than peitiolate and smaller (0.4–0.6 × 0.3–0.5 cm) without clasping; calyx tubes are densely pilose on both surfaces and bear stipitate glands abaxially, in contrast to the slightly pubescent, glandless calyx tubes of *N.gracilis*; and the seed number ranges from 2 to 4 instead of consistently 2. These morphological distinctions are further corroborated by molecular phylogenetic analysis based on 703 single-copy nuclear genes, supporting the recognition of *N.daloushanensis* as a distinct species within the genus.

## ﻿Introduction

The genus *Neillia* D.Don was established by Don (1825) in his Prodromus Florae Nepalensis, where he described two species, *N.thyrsiflora* D.Don and *N.rubiflora* D.Don, both native to Nepal. *Neillia* belongs to the tribe Neillieae within the subfamily Amygdaloideae and represents a small genus (ca. 16 species) of Rosaceae. Since the publication of *N.velutina* Pendry (Pendry 2010)—which was later synonymized under *N.thibetica* Bureau & Franch—the most recent new species description in the genus dates back over 35 years.

The genus *Neillia* belongs to the tribe Neillieae Maxim. (Rosaceae), which comprises three taxonomically challenging genera: *Neillia*, *Physocarpus* (Cambess.) Raf., and *Stephanandra* Siebold & Zucc. (Maximowicz 1879; Schulze-Menz 1964). Over the past three centuries, substantial morphological differences among these genera and their species have resulted in numerous, often contradictory, classification schemes (Oh and Potter 1999). Morphologically, members of the tribe Neillieae are characterized by lobed leaves with persistent or deciduous stipules and ovoid, shiny seeds containing abundant endosperm (Vidal 1963). In 2005, Oh and Potter reconstructed the phylogeny of Neillieae using nucleotide sequences from chloroplast DNA (cpDNA), nuclear ribosomal Internal Transcribed Spacer (nrITS), External Transcribed Spacer (nr ETS) regions, and the second intron of the LEAFY gene. Their phylogenetic analysis of the I-box region from *Neillia* and *Stephanandra* showed that species previously classified as *Stephanandra* were nested within *Neillia*, supporting the merger of the two genera (Oh 2013). These two genera can be distinguished from most other Rosaceae members by their lobed simple leaves, persistent or deciduous stipules, abundant endosperm, and follicles that are sutured and dehiscent along the ventral side (Vidal 1963; Hutchinson 1964; Cullen 1971). Consequently, *Neillia* and *Stephanandra* exhibit high morphological and molecular similarity, though they are typically distinguished by calyx tube shape and seed number.

Dispersion-migration analysis suggested that the nearest common ancestor of the tribe Neillieae likely originated in East Asia and western North America. Biogeographic analysis further indicated that both *Neillia* and *Stephanandra* exhibit their significant diversity in East Asia (Oh and Potter 2005). The genus *Neillia* is primarily distributed in East Asia, occupying subtropical to temperate regions of the Northern Hemisphere. Currently, the genus comprises 16 species, of which 14 are in China, including 10 endemics. China, therefore, represents the diversity center of *Neillia*. Within China, these species are mainly distributed in the southwest, with Yunnan harboring the highest species richness (12 species), followed by Sichuan and Tibet (nine species each), and Guizhou (seven species).

Recent advances in next-generation sequencing (NGS) technologies, coupled with dramatically reduced sequencing costs, have revolutionized phylogenetic reconstruction approaches. Contemporary studies increasingly utilize chloroplast genomes and extensive nuclear gene datasets for robust phylogenetic inference (Zhang et al. 2017; Liu et al. 2019, 2020a, 2020b, 2023a; Wang et al. 2024; Lin et al. 2025a; Peng et al. 2025). Nuclear markers, in particular, are highly valuable due to their biparental inheritance, offering a more comprehensive perspective on evolutionary relationships. This approach has been especially productive in Rosaceae systematic community, facilitating significant progress at both family and generic levels (Xiang et al. 2017; Liu et al. 2022, 2023b; Jin et al. 2023, 2024a, 2024b; Xie et al. 2025). The success of these methods has led to their widespread adoption across diverse taxonomic groups (Liu et al. 2021; Xu et al. 2023; Wei et al. 2024; Lin et al. 2025b), demonstrating their broad utility in contemporary phylogenetic research.

The Dashahe National Nature Reserve in Guizhou is located at the northern edge of Daozhen Gelaozu Miaozu Autonomous County, Guizhou Province. It borders Nanchuan District and Wulong District in Chongqing and is adjacent to the Jinfo Mountain National Nature Reserve (Wang 2024). All of these areas are part of the Dalou Mountains, specifically the southern branch of the eastern section. In July 2024, during a field investigation in Dashahe National Nature Reserve near Mazhuayan, we encountered a creeping liana species. Based on its triangular-ovate leaves with doubly serrate, prominent stipules and follicles with stipitate glandular, we initially assigned it to the genus *Neillia*, although no flowers were observed at the time. Subsequently, on October 26^th^ of the same year, during a follow-up survey at Mazhuayan, we observed the species flowering and fruiting simultaneously. Upon detailed examination, it is clearly distinct from all previously known *Neillia* species, representing a previously unrecorded new species.

## ﻿Materials and methods

### ﻿Morphological characteristics

We collected specimens of this species for morphological description and as voucher specimens. Morphological characteristics were observed and measured from living plants. Comparisons with morphologically similar species were based on their type specimens, and morphological descriptions, photographs and other specimens are obtained through the following channels: Flora of China (http://www.efloras.org/), PPBC (https://ppbc.iplant.cn/), CVH (https://www.cvh.ac.cn/), JSTOR Global Plants (https://plants.jstor.org/).

### ﻿Taxon sampling and DNA sequencing

In this study, we examined 20 accessions of *Neillia*, including 15 newly sequenced specimens (comprising a putative new taxon and the outgroup *Spiraeasalicifolia* L.), supplemented with three published sequences retrieved from National Center for Biotechnology Information (NCBI). Genomic DNA was isolated from silica gel-dried leaf materials and herbarium vouchers using a modified CTAB protocol (Li et al. 2013). Voucher information is provided in Table 1. The raw data were processed using Trimmomatic v.0.39 (Bolger et al. 2014) with default parameters to remove low-quality sequences and adapter contamination. FastQC v.0.12.1 (available at https://www.bioinformatics.babraham.ac.uk/projects/fastqc/) was then used to assess the quality of the processed data and ensure it met the standards for downstream analysis.

**Table 1.** Voucher information for taxa used·in this·study.

Group type	Genus	Species	Data number	Voucher	Data type
ingroup	*Neillia*	*Neilliaaffinis* Hemsl.	HM1395_RNA	s.n.	RNA-Seq
ingroup	*Neillia*	Neilliaaffinisvar.pauciflora (Rehder) J.E.Vidal	ROS798	Anshun Expedition # 902	DGS
**ingroup**	***Neillia***	***Neilliadaloushanensis* M.T.An & Xu Wu, sp. nov.**	**GZAC**	**X.Wu # GZAC**	**DGS**
ingroup	*Neillia*	*Neilliadensiflora* T.T.Yu & L.T.Lu	ROS1109	PE-Xizang Expedition # PE6285	DGS
ingroup	*Neillia*	*Neilliagracilis* Franch.	PE01147903	PE-Xizang Expedition # 12151	DGS
ingroup	*Neillia*	*Neilliahanceana* (Kuntze) S.H.Oh	ROS1009	IBCAS # 40759	DGS
ingroup	*Neillia*	*Neilliaincisa* (Thunb.) S.H.Oh 1	ROS1592	s.n.	DGS
ingroup	*Neillia*	*Neilliaincisa* 2	SRR13065770	s.n.	s.n.
ingroup	*Neillia*	*Neilliaincisa* 3	SRR13065771	s.n.	s.n.
ingroup	*Neillia*	*Neilliaribesioides* Rehder	ROS1419	s.n.	DGS
ingroup	*Neillia*	*Neilliarubiflora* D.Don	ROS1111	PE-Xizang Expedition # 2917	DGS
ingroup	*Neillia*	*Neilliaserratisepala* H.L.Li	ROS1092	PE-Xizang Expedition # PE5719	DGS
ingroup	*Neillia*	Neilliasinensisvar.caudata Rehder	ROS799	S.S.Lan # 389	DGS
ingroup	*Neillia*	*Neilliasinensis* Oliv. 1	SRR26662633	s.n.	s.n.
ingroup	*Neillia*	*Neilliasinensis* 2	XYZ021_RNA	s.n.	RNA-Seq
ingroup	*Neillia*	*Neilliatanakae* Franch. & Sav.	HM803_RNA	s.n.	RNA-Seq
ingroup	*Neillia*	*Neilliathibetica* Bureau & Franch.	ROS1091	PE-Xizang Expedition # PE5713	DGS
ingroup	*Neillia*	*Neilliathyrsiflora* D.Don	ROS960	X.X.Zhou # s.n.	DGS
ingroup	*Neillia*	Neilliathyrsifloravar.tunkinensis (J.E.Vidal) J.E.Vidal	ROS1077	Y.Qin et al. # CWA0142	DGS
ingroup	*Neillia*	*Neilliauekii* Nakai	ROS943	S.M.Zhang # s.n.	DGS
outgroup	*Spiraea*	*Spiraeasalicifolia* L.	ROS583	Weon Ki Paik # s.n.	DGS

### ﻿Data matrix generation and phylogenetic inference

We utilized a previously developed single-copy nuclear (SCN) marker set designed for Amygdaloideae (Jin et al. 2023, 2024a, 2024b) as the reference for phylogenetic analyses. This curated reference dataset comprises 801 SCN genes that were identified using MarkerMiner v.1.0 (Chamala et al. 2015) through comparative analysis of three representative Rosaceae genomes. All subsequent analyses were performed using the Ortho2Web pipeline (available at https://github.com/PhyloAI/Ortho2Web), recently developed by the PhyloAI team (Xu et al. 2023). Briefly, sequence assembly was performed using HybPiper v.2.1.6 (Johnson et al. 2016) with the MarkerMiner-derived references. Initial filtering eliminated paralogous sequences, yielding 706 strictly SCN genes for downstream analyses. Multiple sequence alignments were generated using MAFFT v.7.505 (Nakamura et al. 2018) with the --localpair --maxiterate 1000 parameters. To ensure alignment quality given variable sequencing coverage, we performed rigorous filtering using trimAL v.1.4 (Capella-Gutiérrez et al. 2009), removing alignment columns containing gaps in more than 20% of sequences or showing similarity scores below 0.001. Following rigorous filtering to remove paralogous sequences and low-quality loci, we identified 703 high-quality SCN genes that were retained for phylogenetic reconstruction.

We employed concatenation-based approaches for the phylogenetic inference. These 703 high-quality SCN genes were compiled into supermatrices using AMAS v.1.0 (Borowiec 2016). Optimal partitioning schemes and nucleotide substitution models were determined using PartitionFinder2 (Lanfear et al. 2017), with linked branch lengths and model selection based on the corrected Akaike Information Criterion (AICc). The rcluster algorithm (Lanfear et al. 2014) was implemented during this process to handle the SCN gene dataset. Maximum Likelihood (ML) analyses were conducted in IQ-TREE2 v.2.2.6 (Minh et al. 2020) using the optimal partitioning schemes identified by PartitionFinder2. IQ-TREE2 was employed with the parameters “-B 1000 -alrt 1000” to evaluate tree robustness through 1000 rapid bootstrap replicates and approximate likelihood ratio tests (ALRT).

## ﻿Taxonomic treatment

### ﻿ Neillia daloushanensis

Taxon classificationPlantaeRosalesRosaceae

M.T.An & Xu Wu sp. nov.

D52E5492-689D-5CEB-808E-285826A9DEB2

urn:lsid:ipni.org:names:77367214-1

Figs 1 , 2 , 5

#### Type.

China • Guizhou Province, Daozhen Gelaozu Miaozu Autonomous County, Dashahe National Nature Reserve, Mazhaoyan, 29°11'N, 107°27'E, alt. 1910 m, 26 October 2024, *Ming-tai An*, *Xu Wu*, *Yu-cai Feng*, *Hua-kai Zou*, *Jin-xiong Ba*, GZAC-DSH-001 (***holotype***: GZAC!; ***paratype***: PE!).

#### Diagnosis.

The new species is morphologically most similar to *Neilliagracilis* Franch., but differs notably in several characters. *N.daloushanensis* is a creeping liana with stout and quadrangular branchlets, whereas *N.gracilis* is not. Its leaves are approximately twice as long and wide as those of *N.gracilis*. The stipules are large and wide, broadly ovoid, sessile, and clasping, measuring 0.8–1.3 cm in length and 0.6–1.1 cm in width. The calyx tube is densely pilose on both surfaces and bears stipitate glands abaxially. Seeds number 2–4 (vs. 2 in *N.gracilis*, details refer to Table 2).

**Table 2.** Morphological comparison of species of *N.daloushanensis* and its relatives.

Character	N.daloushanensis	N.gracilis	N.affinis	N.sinensis
Growth form	Lianas, rhizomes woody	Subshrubs low	Erect shrubs	Erect shrubs
Stem	0.5–1.4 m long, stout	Less than 0.5 m tall, slender	Ca. 2 m tall, slender	Ca. 4 m tall, slender
Leaf size	5–9 × 3–6 cm	2.5–3.5 × 2–3 cm	3.5–6.8 × 3–5 cm	5–11 × 3–6 cm
Leaf margin	Obtuse doubly serrate	Sharply doubly serrate	Serrate	Sharply doubly serrate
Leaf surface pubescence	Both surfaces are pubescent along veins, the rest are pubescence less	Both surfaces are sparsely pubescent or subglabrous	Both surfaces are glabrous or subglabrous	Glabrous on both surfaces or abaxially pubescent in vein axils
Stipules	0.8–1.3 × 0.6–1.1 cm, herbaceous, wide-ovate, margin undulate or obtusely crenate, sessile, apex blunt, clasping	0.4–0.6 × 0.3–0.5 cm, herbaceous, ovate or triangular-ovate, margin serrate, stipule petiole, without clasping	Ca. 0.4 cm, membranous, long ovate to linear-lanceolate, without clasping	0.8–1 cm long, membranous, anceolate to ovate-lanceolate, margin entire, without clasping
Racemes length	Ca. 2.4 cm	1–1.8 cm	3–8 cm	4–9 cm
Peduncle and pedicels	Pubescent	Subglabrous	Pubescent	Glabrous
Calyx tube	Densely pilose on both surfaces, abaxially stipitate glandular	Abaxially slightly pubescent, without glands	Abaxially densely pubescent and stipitate glandular	Abaxially glabrous or glandular
Seeds	2–4	2	4–6 (10)	4–5

**Figure 1.**
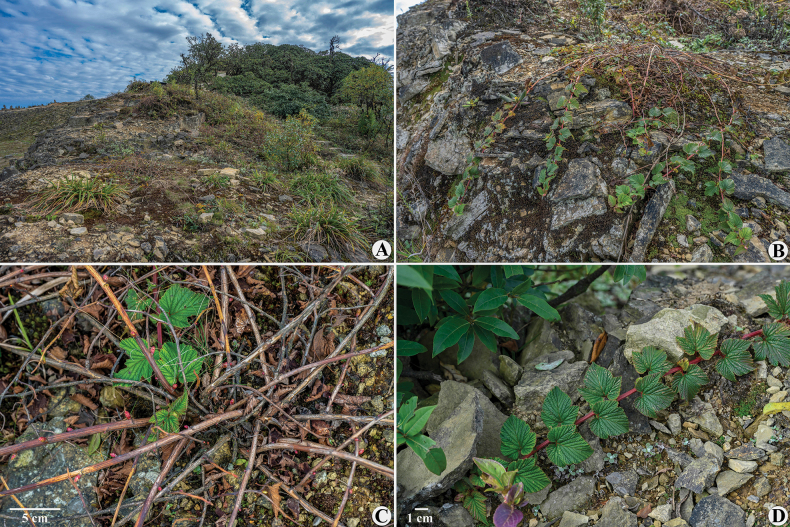
*Neilliadaloushanensis*. A. Habitat; B. Plant; C. Stem base; D. Stems and leaves.

**Figure 2.**
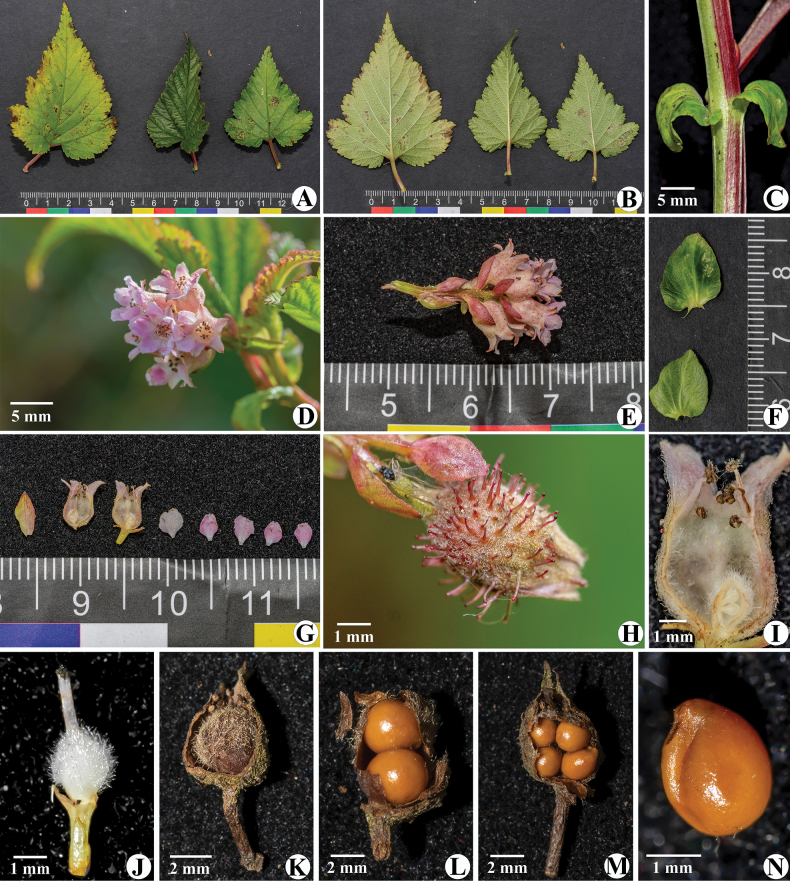
*Neilliadaloushanensis*. A. Leaves from above; B. Leaves from below; C, F. Stipules; D. Flowers; E. Raceme; G. Dissected flower; H. Stipitate glands; I. Calyx tube; J. Ovary; K. Fruit; L, M, N. Seeds.

#### Description.

***Creeping lianas***, rhizomes woody. ***Branchlets*** stout, angular, glabrous, 0.5–1.4 m long. ***Leaves*** blade ovate or triangular-ovate, 5–9 × 3–6 cm, apex acuminate or long acuminate, base heart-shaped, margin obtuse doubly serrate and irregular 3–5-lobed, rarely indehiscent, pubescent on both surfaces along veins. Petiole 1.2–2.3 cm long, sparsely pilose. ***Stipules*** large and wide, broadly ovoid, sessile, clasping, 0.8–1.3 × 0.6–1.1 cm, apex blunt, margin wavy-toothed, rarely entire, ciliate. ***Terminal raceme*** with 4–11 flowers, ca. 2.4 cm long. Bracts ovate, margin with ciliate. Pedicel ca. 0.2 cm, pilose. ***Flowers*** ca. 0.5 cm in diameter. ***Calyx tube*** bell-shaped, 0.3–0.4 cm long, densely pilose on both surfaces, covered with abaxially stipitate glandular. ***Sepals*** triangular-ovate, 0.2–0.3 cm long, apex tapering, margin entire, slightly pilose on both surfaces. ***Petals*** obovate, white or pinkish, ca. 0.3 × 0.3 cm, apex emarginate, ciliate. ***Stamens*** ca. 20, inserted at the edge of the calyx tube, not exceeding the sepals in height. ***Ovary*** densely villous, with 2–4 ovules. ***Follicles*** hidden in the persistent calyx tube. ***Seeds*** 2–4, bright brown, with raised seed ridges.

#### Distribution and habitat.

**Figure 3.**
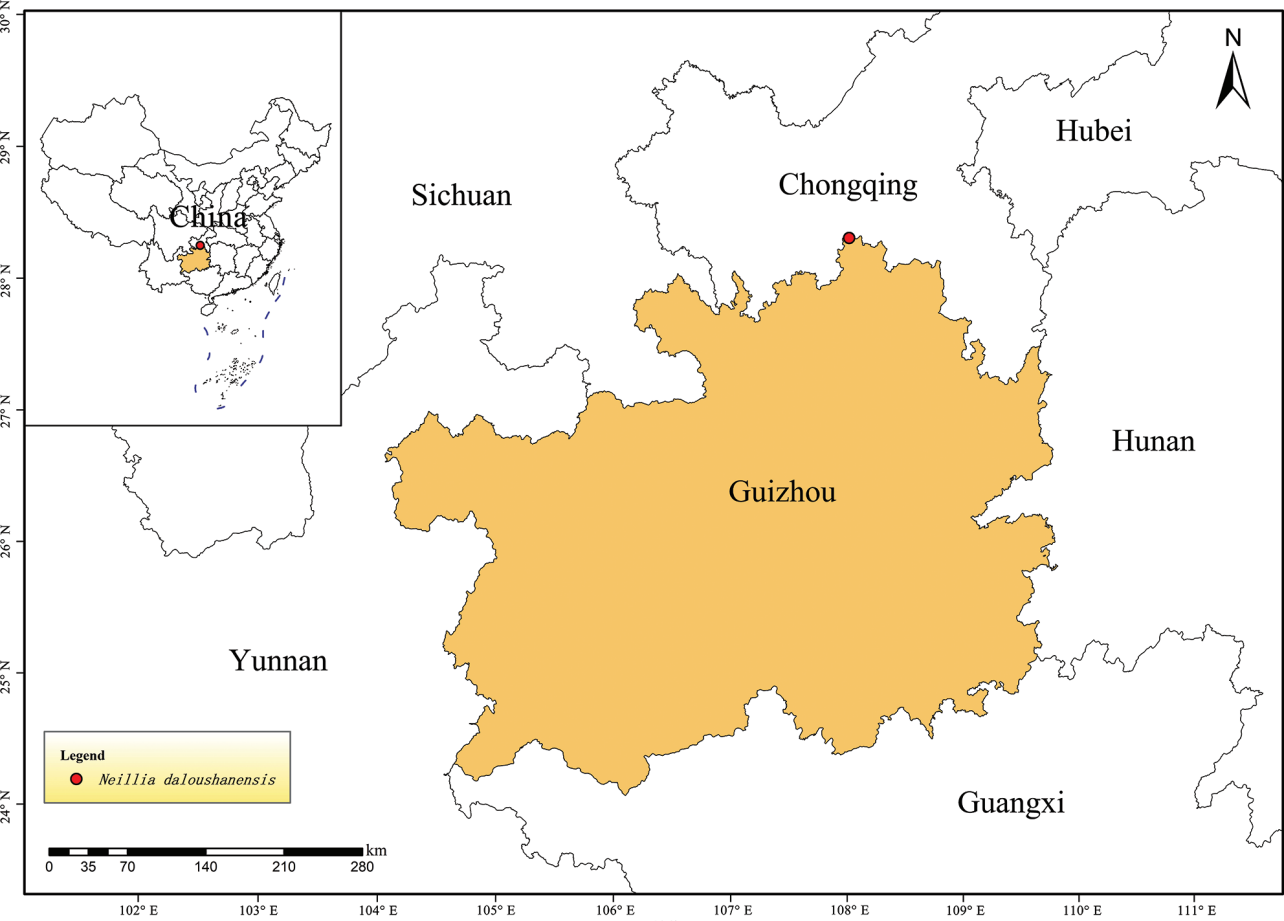
Geographical distribution of *Neilliadaloushanensis*.

This species is currently known only from Dashahe National Nature Reserve, Daozhen Gelaozu Miaozu Autonomous County, Guizhou Province, where it grows on bare high-altitude land or at the edges of shrubs at an altitude of ca. 1900 m (Fig. 3). It may also occur in adjacent areas of Chongqing Province.

#### Phenology.

The flowering and fruiting period is from September to December.

#### Etymology.

“daloushanensis” specifically refers to the Dalou mountain range.

#### Local name.

Simplified Chinese: 大娄山绣线梅.

#### Conservation status.

We investigated potential habitats in the surrounding area and found that *Neilliadaloushanensis* primarily grows on sandy-gravel soil with ample light and good drainage. The soil in these habitats is relatively poor, and the species is absent from adjacent shrublands characterized by deep humus layers. Although *N.daloushanensis* occasionally occurs at forest edges, its growth there is noticeably poorer than on exposed bare ground. Due to the limited extent of suitable bare land, the estimated population size is fewer than 50 individuals. Additionally, a review of image and specimen databases from regions with similar habitats and elevations revealed no other known populations. However, as a comprehensive investigation of the population status of *N.daloushanensis* has not yet been conducted, we recommend classifying it as Data Deficient “DD” according to the IUCN criteria (IUCN 2022).

#### Phylogenetic affiliation.

**Figure 4.**
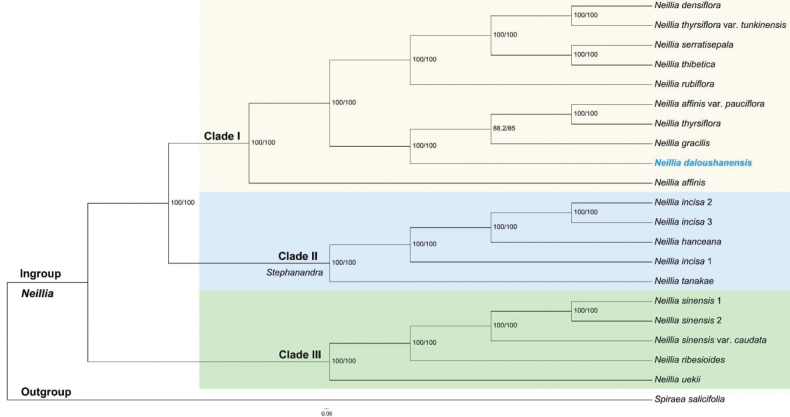
Phylogenetic tree of *Neillia* estimated using the Maximum Likelihood (ML) algorithm with IQ-TREE2 based on 703 single-copy nuclear (SCN) genes. Numbers on the branches indicate ML bootstrap values.

The phylogenetic analysis reveals that *N.daloushanensis* forms a distinct clade sister to a group comprising N.affinisvar.pauciflora, *N.thyrsiflora*, and *N.gracilis*, rather than clustering exclusively with its morphologically similar species, *N.gracilis* (Fig. 4). Morphologically, only *N.daloushanensis* is a semi-lignified creeping liana, which can also be well distinguished by the large and wide stipules, densely pilose calyx tube on both surfaces and seeds 2–4.

**Figure 5.**
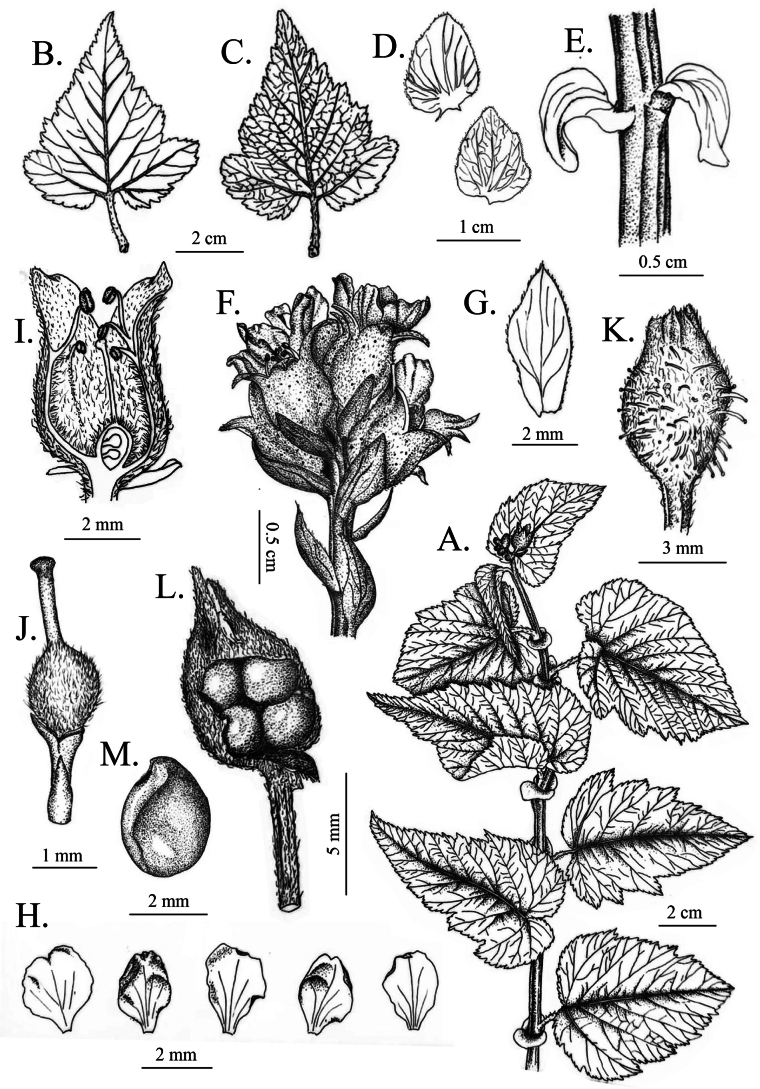
*Neilliadaloushanensis*. A. Plant; B. Leaves from above; C. Leaves from below; D, E. Stipules; F. Raceme; G. Flower bract; H. Petals; I. Calyx tube; J. Ovary; K. Stipitate glands; L. Fruit; M. Seed.

## ﻿Discussion

Based on morphology, *N.daloushanensis* can be easily distinguished from all other species of the genus *Neillia*. Previously, species in this genus were recorded exclusively as erect shrubs. The discovery of *N.daloushanensis*, a creeping liana (Fig. 1B–D), expands the known morphological diversity of *Neillia* and enriches its species diversity. Notably, the large, broad stipules and clasping stems of *N.daloushanensis* are distinct features that distinguish it from other plants. Their morphological traits appear closely linked to its habitat: *N.daloushanensis* grows on exposed bare ground at mountain summits. Its creeping stems likely represent an adaptation to strong winds and facilitate heat absorption. Therefore, *N.daloushanensis* also differs from other species of the genus *Neillia* in its ecological preferences.

Phylogenetic results strongly support *Neilliadaloushanensis* as an independent branch nested within a larger clade that includes *N.gracilis*. Both morphological and molecular evidence indicate a close relationship between these species. From an evolutionary perspective, the specialized growth habits of *N.daloushanensis* may represent an independent adaptive radiation event within the genus *Neillia*. The phylogenetic placement of *N.daloushanensis* within an existing clade suggests a relatively recent adaptive divergence. Its geographically isolated distribution, restricted to the Dalou Mountains, may be related to the complex geologic history and refugial dynamics. Moreover, habitat heterogeneity in this area may have facilitated the speciation and ecological niche differentiation of *N.daloushanensis*.

## Supplementary Material

XML Treatment for Neillia daloushanensis

## ﻿Additional information

### Use of AI

No use of AI was reported.

### Author ORCIDs

Xu Wu https://orcid.org/0009-0004-7935-6057

Dai-Kun Ma https://orcid.org/0009-0005-5523-508X

Ming-Tai An https://orcid.org/0000-0003-3886-0287

Jiang-Hong Yu https://orcid.org/0000-0003-1765-8557

### Data availability

All of the data that support the findings of this study are available in the main text or Supplementary Information.
